# Oxidative stress levels and oral bacterial milieu in the saliva from pregnant vs. non-pregnant women

**DOI:** 10.1186/s12903-020-01230-3

**Published:** 2020-09-03

**Authors:** Madhu Wagle, Purusotam Basnet, Åse Vartun, Tordis A. Trovik, Ganesh Acharya

**Affiliations:** 1 Women’s Health and Perinatology Research Group, Department of Clinical Medicine, Faculty of Health Sciences, UiT The Arctic University of Norway, N– 9037 Tromsø, Norway; 2 Department of Obstetrics and Gynaecology, University Hospital of North Norway, Tromsø, Norway; 3 Department of Community Medicine, Faculty of Health Sciences, UiT The Arctic University of Norway, Tromsø, Norway; 4 Division of Obstetrics and Gynaecology, Department of Clinical Science, Intervention and Technology, Karolinska Institutet, Stockholm, Sweden

**Keywords:** Oral health, Bacterial milieu, Oxidative stress (OS), Total anti-oxidant capacity (TAC), Malondialdehyde (MDA), Saliva, Pregnancy

## Abstract

**Background:**

Saliva plays a significant role in maintaining oral health and oral bacterial milieu. Difference in oxidative stress (OS) levels in saliva in conjunction with bacterial load between pregnant and non-pregnant women has not been studied previously. We hypothesized that the physiological changes in pregnancy alter oral bacterial milieu by promoting growth of *Streptococcus mutans* (SM) and *Lactobacillus* (LB), and increase OS in saliva. The aim of this study was to measure and compare the oral bacterial milieu, OS and total anti-oxidative capacity (TAC) in the saliva of pregnant and non-pregnant women.

**Method:**

In this cross-sectional study, we assessed oral bacterial milieu by culturing the SM and LB by using commercial kits, TAC by measuring 2, 2′-Azino-Bis-3-Ethylbenzothiazoline-6-Sulfonic Acid (ABTS) free radical scavenging activity spectrophotometrically and OS levels by measuring malondialdehyde (MDA) levels with commercial kits in the saliva of pregnant women (*n* = 38) at 18–20 weeks of gestation, who were compared with age-matching healthy non-pregnant women (*n* = 50).

**Results:**

*Streptococcus mutans* were found to be more abundant in the saliva of pregnant women compared with non-pregnant women (*p* = 0.003) but the difference was not significant for the LB (*p* = 0.267). TAC was found to be 46% lower in pregnant women’s saliva compared to non-pregnant women [optical density (OD) measured at 731 nm as 0.118 ± 0.01 vs. 0.063 ± 0.02; *p* < 0.001]. OS, expressed as saliva MDA levels, was found to be 16% higher in pregnant women compared to non-pregnant women (1.07 nM MDA vs. 0.92 nM MDA; *p* = 0.023).

**Conclusion:**

Pregnancy has an adverse impact on oral bacterial milieu as demonstrated by increased colonization with *Streptococcus mutans* together with higher OS levels and decreased TAC levels in saliva. This emphasizes the importance of improved oral hygiene and provision of oral healthcare services during pregnancy care.

## Background

Saliva is an important aqueous oral fluid that contributes to the maintenance, preservation, protection and healing of oral tissues along with other functions such as helping in speech, lubrication, taste perception and digestion. Saliva is also considered the “mirror of body”, and in recent years is being widely used as a tool to screen and diagnose diseases, monitor disease progression, measure drug levels etc. due to its ease of collection and abundance of biomarkers present [1–8]. In addition to this, saliva also has a role in immunological and enzymatic defence mechanisms against certain microorganisms´ antioxidant system and the body’s overall oxidative stress (OS) is expressed in saliva [8, 9].

Oxidative stress is the state of an imbalance between oxidants and anti-oxidant systems leading to and causing potential damage of cellular physiology [10]. OS is recognized as a major contributor to several oral conditions, such as salivary gland dysfunctions, xerostomia, periodontitis, precancerous lesions and oral carcinogenesis. Malondialdehyde (MDA) is an indicator of OS as it is one of the final products of lipid peroxidation reaction resulting from increased levels of reactive oxygen species (ROS). Higher MDA levels and lower salivary anti-oxidant activity have been reported in patients suffering from periodontitis [11]. In recent years, studies have highlighted that OS may have an influence on the human reproductive system [12–14]. Increased vulnerability to OS during pregnancy may predispose to spontaneous abortion, recurrent pregnancy loss, pre-eclampsia, and gestational diabetes [15, 16]. Offenbacher et al. were the first, in 1996, to point out that periodontal disease is a potential risk factor for preterm birth [17]. Since then, the link between periodontal infections and preterm birth has been one of the frontiers in dental research. However, recent epidemiological studies largely support a strong association between poor oral health and adverse pregnancy outcomes, while some controversy still remains [18].

Hormonal fluctuation and immunological changes are physiological phenomena during pregnancy that may predispose to poor oral health. Although poor oral health has been shown to be associated with adverse pregnancy outcomes, preventive dentistry and oral health care is neither the focus nor a part of routine prenatal care in most countries, including Norway. OS in the blood samples of pregnant women was found to be higher than that of healthy non-pregnant women [14]. Therefore, OS measurement in the saliva of pregnant women, together with the assessment of oral bacterial milieu, could be important to understand the cross-link between OS, oral health, and pregnancy outcome.

*Streptococcus mutans* (SM) and *Lactobacillus* (LB) are reported as the major culprit causing dental caries in humans [19–22]. Therefore our main focus in this study is on these bacterial species. We hypothesized that the physiological changes in pregnancy alter oral bacterial milieu by promoting growth of SM and LB, and increase OS in saliva. The objective of this study was to measure and compare the bacterial milieu, OS and total anti-oxidative capacity (TAC) in the saliva of pregnant and non-pregnant women.

## Methods

This cross-sectional study was part of an ongoing prospective study on oral health in pregnancy conducted at the University Hospital of North Norway, Tromsø, Norway. Saliva samples collected consecutive from 38 healthy pregnant women and 50 healthy non-pregnant women were used for determining the bacterial milieu and OS levels. Women were recruited to the study when they attend the hospital for routine second trimester ultrasound screening at 18–20 weeks. Inclusion criteria were: age > 18 years, low risk singleton pregnancy, no previous history of any pregnancy-associated complications such as preeclampsia, preterm birth or gestational diabetes, and absence of any preexisting medical condition that may have an impact on the course and outcome of pregnancy. Pregnant women who were not willing to participate, could not communicate in Norwegian or English, and those who have been diagnosed to have a fetus with a chromosomal or structural fetal anomaly and did not plan to continue their pregnancy, were excluded. Age matched non-pregnant healthy women of reproductive age were recruited among women working at the University of Tromsø or the University Hospital of North Norway, Tromsø. A history of any acute or chronic illness requiring regular medical treatment excluded participation. All participants were informed about the study in advance and a written consent was obtained from all participants. The study was approved by the Regional Committee for Medical and Health Research Ethics - North Norway (Ref no: 2012/633/REK nord).

### Collection of saliva samples

Saliva samples for both groups were collected using identical methods. In brief, paraffin wax stimulated saliva samples were obtained by expectorating in disposable cups. For oral bacterial milieu, two main bacteria, SM and LB, were tested. For OS study, 1.8 ml of saliva was collected in cryo-tubes vials and stored at -70 °C until samples were analyzed. For the TAC and OS analysis, samples were stored in a refrigerator at 4 °C for 1 day before analysis was performed. On the day of analysis, samples were kept at room temperature for 2 h and centrifuged at 10000 x g for 10 min to remove cell debris and supernatant that was collected for further analysis. Storing-procedures and laboratory analyses were processed according to the kit manufacturer’s instructions.

### Bacterial milieu assessment in saliva

Oral bacterial milieu was assessed by the cultivation and development of bacterial colony forming units (CFU) of two main bacteria, SM and LB, using commercial kits Dentocult® LB (kit for LB), and Dentocult® SM Strip mutans (kit for SM) (Orion Diagnostica Oy, Espoo, Finland). Women were requested to chew a paraffin pellet to stimulate the secretion of saliva and promote transfer of SM from tooth surfaces into the saliva. A round-tipped test strip supplied in the kits was pressed against the saliva on the woman’s tongue. The strip was placed in the cap of the vail containing culture broth and was recapped in the vail. The vial was loosely capped and incubated at 37 °C and 5% CO_2_ for 48 h. Results were interpreted by scoring as 0, 1, 2, and 3 for 0, < 10^5^, 10^5^–10^6^ and > 10^6^ CFU/mL, respectively, by comparing to the template reader provided in the kits. In case of LB culture, the test strip was thoroughly made wet by saliva, fixed in the cap and fitted in the vials containing culture broth. It was then incubated for 4 days at 37 °C and 5% CO_2_. Results were interpreted scoring as 0, 1, 2, 3 and 4 for 0, 10^3^, 10^4^, 10^5^ and 10^6^ CFU/mL, respectively, by comparing to the template reader provided in the kits. Results are expressed as the percentage among pregnant and non-pregnant women based on the development of bacterial CFU.

### Measurement of Total Antioxidant Capacity (TAC) in saliva

Total antioxidant capacity (TAC) in the saliva was expressed by measuring 2,2′-azino-bis(3-ethylbenzothiazoline)-6-sulfonic acid diammonium salt (ABTS) free radical scavenging activity [23]. In brief, a dark green color of ABTS free radicals was generated by mixing 2 mL of each of the solutions of ABTS (7.4 mM) and potassium peroxodisulfate (2.6 mM) for 24 h. Both chemicals, ABTS and potassium peroxodisulfate were purchased from Sigma-Aldrich, Oslo. The reaction mixture was diluted to 100 mL with distilled water as a working solution ABTS free radical. Optical density (OD) of the working ABTS radical solution was approx. 0.5 to 0.6. Supernatant of saliva samples were used for both groups. Reactions were carried out by mixing 450 μL of working solution of ABTS radical and 50 μL supernatant part of saliva followed by incubating for 30 min in darkness. The change in the green color of ABTS free radicals scavenged by the antioxidants present in saliva fluid was measured for OD using spectrophotometric methods (Agilent Technologies Deutschland GmbH, Waldbronn, Germany) at 731 nm. Higher OD_731_ value represents lower level of TAC. Water soluble vitamin C (Sigma-Aldrich) was used as a standard and TAC was quantified as μg/mL vitamin C equivalent level, representing the total antioxidant capacity (TAC) with the help of standard curve and regression equation (*R*^*2*^ = 0.9331 and *y* = − 0937x + 0.7357).

### Oxidative stress levels in saliva by malondialdehyde (MDA) assay

We measured saliva MDA content using a commercially available MDA Assay Kit (Sigma-Aldrich, Lipid Peroxidation MDA Assay Kit) following the instructions provided by the supplier; MDA levels are expressed as OS levels [24]. In brief, a mixture of 100 μL saliva fluid is diluted with 200 μL buffer provided in the kit. The saliva sample in buffer and Thiobarbituric acid (TBA) solution, 600 μL each, were mixed thoroughly and incubated at 95 °C for 60 min. Of the reaction mixture, after cooling in ice, 150 μL was transferred to a 96 well microplate in duplicates and absorbance was measured flurometrically (Epoch Microplate, BioTek Instrument, Vermont, USA) by measuring fluorescence intensity (λ_ex_ = 532/ λ_em_ = 553). The MDA levels in the saliva were calculated by the MDA standard provided in the kit with the help of standard curve and regression equation (*R*^*2*^ = 0.9903 and *y* = 728.95x + 111.6).

### Statistical analysis

The sample size required a detection of 15% difference in the OS level between pregnant and non-pregnant women, with 80% power at an alpha of 0.05, calculated to be at least 38 individuals per group on the basis of mean MDA level and standard deviation reported in the saliva of 25 healthy female controls in a previous report [25] using an online sample size calculator [26].

Data analysis was performed using IBM SPSS Statistics for Windows, Version 25.0. (IBM Corp, Armonk, NY). Data are presented as mean (SD) or median (IQR) as appropriate. Frequency tables were made and comparison between the pregnant and non-pregnant groups was carried out by conducting χ^2^ (chi-squared) test for categorical variables with Bonferroni adjustment when appropriate, and an independent sample t-test for parametric continuous variables. The strength of correlation between two continuous variables was assessed by Pearson’s correlation coefficient. A *p*-value of < 0.05 was considered statistically significant.

## Results

Data from a total of 38 pregnant and 50 non-pregnant women were included in the analysis. The median (IQR) age of the pregnant and non-pregnant groups were 31.5 (5.8) and 30 (8) years, respectively.

### Oral bacterial milieu

**Fig. 1:**
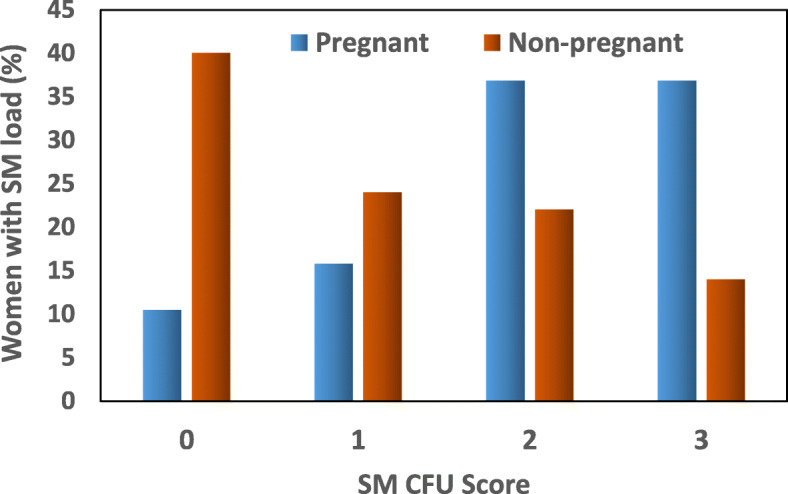
Comparison of *Streptococcus mutans (SM)* bacterial milieu in the saliva of pregnant and non-pregnant women. The bars in the diagram represent the percentage of women scoring 0, 1, 2, and 3 based on the number of colony-forming units (CFU) of bacteria identified after culture, i.e. 0, 10^5^, 10^5^–10^6^ and > 10^6^ CFU/mL, respectively. *Pregnant vs. non-pregnant group; *p* < 0.05 (chi-squared test). **Pregnant vs. non-pregnant group (difference was only significant (*p* < 0.05.) between subgroups with SM score 0 and 3 (chi-squared test with Bonferroni adjustment)

**Fig. 2:**
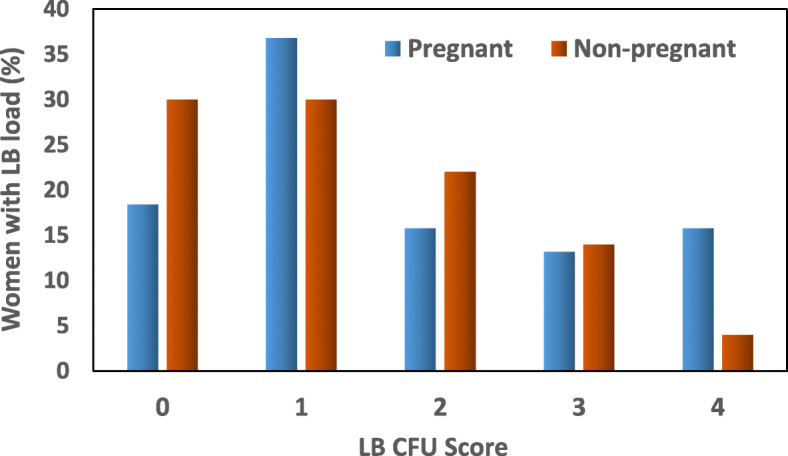
Comparison of *Lactobacillus (LB)* bacterial milieu in the saliva of pregnant and non-pregnant women. The bars in the diagram represent percentage of women scoring 0, 1, 2, 3 and 4 based on the number of colony-forming units (CFU) of bacteria identified after culture, i.e. 0, 10^3^, 10^4^, 10^5^ and 10^6^ CFU/mL, respectively. There were no significant differences between pregnant and non-pregnant groups

Figures 1 and 2 show the distribution of salivary levels of SM and LB in pregnant and non-pregnant women.

The SM bacterial milieu profiles as compared between the groups of pregnant and non-pregnant women are shown in Fig. 1. SM colonies were found to be more abundant and significantly higher in the saliva of pregnant compared to non-pregnant woman (χ^2^ statistic = 13.984; *p* = 0.003). The majority of pregnant women were highly colonized with SM compared to non-pregnant women. In the group of pregnant women, 73.6% were found to have developed 10^5^ or more CFU/mL in the culture.

The LB bacterial milieu profiles as compared between the groups of pregnant and non-pregnant women are shown in the Fig. 2. The LB bacterial colonies were abundant in the saliva of pregnant women, but not significantly higher compared to non-pregnant women (χ^2^ statistic = 5.208; *p* = 0.266). Among pregnant women, 15% developed 10^6^ CFU/mL after the culture compared to 4% among the non-pregnant women. The majority of the non-pregnant women showed 10^3^ or less CFU/mL in their saliva. Approximately 45% of pregnant women showed 10^4^ or more CFU/mL of LB bacterial colonies compared to 40% in non-pregnant women.

### Total Anti-oxidative Capacity (TAC) in saliva

**Fig. 3:**
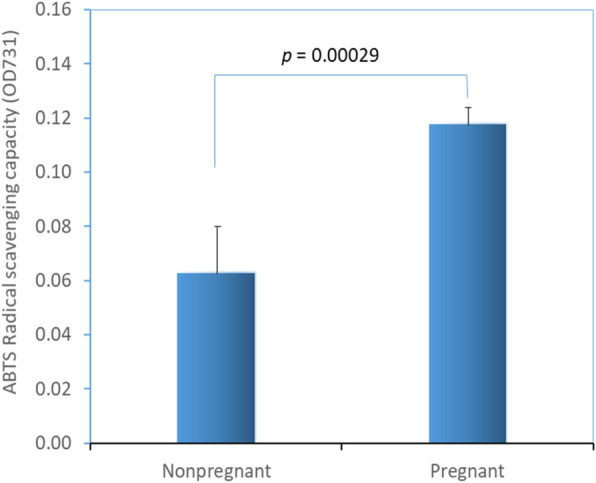
Total anti-oxidant capacity (TAC) in saliva of pregnant (*n* = 38) and non-pregnant (*n* = 50) women. The results are shown as the mean (SD) values for each group expressed as ABTS radical scavenging capacity measured spectrophotometrically as optical density at 731 nm (OD_731_). Difference between groups was highly significant (*P* = 0.00029; independent sample t-test)

The results of TAC in the saliva of pregnant and non-pregnant women are shown in Fig. 3. The average ABTS radical scavenging capacity in the saliva of pregnant women were 46% lower compared to that of non-pregnant women (OD_731_: 0.118 ± 0.01 vs. 0.063 ± 0.02; *p* < 0.001). TAC levels in the saliva of pregnant women (*n* = 38) and non-pregnant women (*n* = 50) were calculated as 6.59 μg/mL and 7.17 μg/mL vitamin C equivalent, respectively.

### Oxidative Stress (OS) levels in saliva

**Fig. 4:**
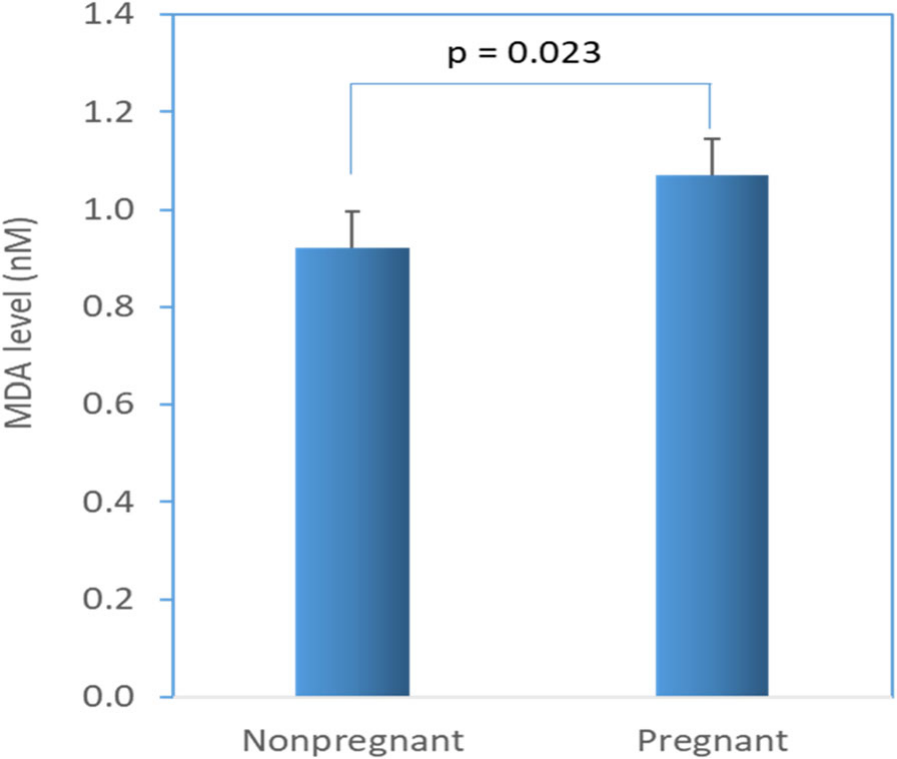
Oxidative stress level in saliva of pregnant and non-pregnant women. The results are shown as mean (SD) of malondialdehyde (MDA) values for pregnant (*n* = 38) and non-pregnant (*n* = 50) groups. The difference between groups was significant (*p* = 0.023; independent sample t-test)

The results of MDA contents in the group of pregnant and non-pregnant women are shown in Fig. 4. The OS levels are expressed as the MDA content present in the saliva. The pregnant women had a 16% higher level of OS in their saliva compared to the non-pregnant women. The average OS levels, expressed as MDA levels in the saliva of pregnant women (*n* = 38) and non-pregnant women (*n* = 50) were 1.07 nM and 0.92 nM; *p* = 0.023), respectively.

### Oxidative Stress (OS) and Total Antioxidant Capacity (TAC) in saliva in relation to oral bacterial load

The results of correlation analysis between OS or TAC in the saliva and oral bacterial load expressed as colonization by SM and LB after 48 h or 96 h of culture for both pregnant and non-pregnant group are presented in Table 1. No statistically significant correlations were found. (Table 1).

**Table 1:** Correlation between oxidative stress (OS) or total antioxidant capacity (TAC) with bacterial load (SM or LB) among the groups of pregnant and non-pregnant women

Variables	Groups	*Pearson r*	*p*-value
*ABTS*			
SM	Pregnant	0.20764	0.2386
LB		0.10593	0.5509
SM	Non-pregnant	0.10082	0.4860
LB		0.11193	0.4389
*MDA*			
SM	Pregnant	0.11009	0.5105
LB		0.29464	0.0725
SM	Non-pregnant	0.17653	0.2200
LB		0.01280	0.9296

2,2′-azino-bis(3-ethylbenzothiazoline)-6-sulfonic acid diammonium salt (ABTS), *Lactobacillus* (LB), malondialdehyde (MDA), *Streptococcus mutans* (SM)

## Discussion

Saliva, in addition to minerals, mucus, antibacterial compounds and enzymes [27], also carries a portion of antioxidants, such as vitamin C and vitamin E. Saliva has a pivotal role in maintaining the microbial taxa in the oral cavity, as well as oral health. However, there are limited studies on the effect of pregnancy on oral bacterial milieu and OS. In this study, we explored the differences in the oral bacterial milieu and OS levels in saliva between pregnant and non-pregnant women, demonstrating that pregnancy may adversely affect oral health by promoting abnormal bacterial growth and increasing OS levels in the saliva.

More than 700 microbial taxa are found in the oral cavity [28, 29]. Several microbial species reported in the oral cavity are known to cause intrauterine infection without being found in the urogenital tract [30–32]. Surprisingly, a study on microbiomes has demonstrated that microbes found in term placenta are similar to oral rather than vaginal microbes [33]. There are two main hypothetical routes for oral microbes to cause intrauterine infection: either hematogenous dissemination, particularly with periodontal disease [34], or colonization of the vaginal tract with microbes from the oral cavity during receptive oral sex [35]. Periodontal disease is associated with a two to seven-fold increase in preterm birth [36, 37] and a link between maternal periodontal disease and preeclampsia has been suggested [38]. A large multicenter trial comparing women treated for periodontal disease at < 21 weeks vs. post-delivery found a trend for reduced early preterm birth < 32 weeks [39]. These scientific findings demonstrate that oral microbiota are associated with pregnancy outcome. Furthermore, studies have demonstrated that pregnant women are at high risk of caries development [21]. Among oral microbes, SM and LB are most strongly associated with the dental caries [21, 22]. SM is not found anywhere else except in human oral cavity [40]. Based on this background, we measured oral bacterial milieu of SM and LB in the saliva of pregnant women and compared with non-pregnant women. Among pregnant women, only four (10.5%) women out of 38 did not show the presence of SM in the saliva, whereas among non-pregnant women, 20 (40%) women out of 50 did not have SM in their oral cavity. A significantly higher percent of pregnant women participating in our study were colonized with SM (almost 89%), which is in line with previous studies conducted on pregnant populations where 100% of women were found to be infected by SM [21, 41]. Both SM and LB were found to be abundant in pregnant women’s saliva although the difference between pregnant and non-pregnant groups was not statistically significant for LB.

Increased bacterial colonization of oral cavity could be connected with TAC and OS in saliva. Therefore, we measured TAC and OS in the saliva of the pregnant women and compared these with non-pregnant women. We found lower TAC and higher OS in pregnant women compared to non-pregnant women. However, the oral bacterial load of SM and LB did not significantly correlate with TAC or OS in either group. It is not clear to us whether the decreased levels of TAC and increased levels of OS makes a favorable condition for bacterial milieu in the pregnant women or whether increased bacterial growth leads to decreased TAC and increased OS. In general, for the purpose of counteracting and minimizing the damage produced by the ROS, living cells operate antioxidant systems such as enzymes, macromolecules and an array of small molecules. Low levels of TAC could be a sign of increased OS and increased potential for oxidative damage [42, 43].

We measured a 46% lower value of anti-oxidant capacity in the saliva of pregnant women compared to that of non-pregnant women. Saliva contains vitamin C and vitamin E which enhance the total anti-oxidative system of the oral cavity. Vitamin C concentration in saliva has been reported to be 6 to 10 μg/mL [44]. In our study, TAC levels in the saliva of pregnant women and non-pregnant women were calculated to be 6.59 μg/mL and 7.17 μg/mL of vitamin C equivalent, respectively. Expression of total antioxidants in the saliva correlates with the vitamin C level in saliva. We did not measure vitamin C concentration directly in the saliva. However, we measured ABTS radical scavenging activity of saliva, and the majority of the TAC effect is due to the vitamin C reflecting the range of saliva vitamin C concentration. Vitamin C plays an important role in maintaining the integrity of teeth and also contributes to non-enzymatic anti-oxidant defense. Decreased serum and/or salivary vitamin C levels correlate with dental caries [44]. Therefore, decreased TAC may predispose women to poor oral health during pregnancy.

OS occurs when the production of reactive oxygen species (ROS) overwhelms the anti-oxidants that conquer them [45–47]; the net result is damage to cellular structures such as DNA, protein and lipids. ROS are constantly formed within the cells as a by-product of metabolic processes, and a low to moderate level of ROS is physiological and serves as signaling molecules [45, 46]. The levels of ROS and OS directly relate to the corresponding metabolites. MDA is one of the cellular lipids metabolites generated by the ROS reaction. Hence, increased levels of MDA indicate higher levels of OS. We measured MDA contents in the saliva of pregnant and non-pregnant women in order to determine the level of OS. In this study, OS was found to be 16% higher in the saliva of pregnant women compared to non-pregnant women (*p* = 0.023).

Previous studies have described the association between poor periodontal health and risk of preterm birth and low birth weight [36, 37, 48]. However, in a recent systematic review on dental caries and preterm birth, we found that dental caries was not significantly associated with preterm birth [49]. Whether the level of OS in the oral cavity rather than specific disease categories would be more predictive of adverse pregnancy outcomes needs further investigation.

One major strength of our study is that the pregnancy associated change in oral bacterial milieu was validated not only by the assessment of colonization of oral cavity by SM and LB, but also by the measurement of TAC and OS levels in the saliva demonstrating direct consequences of altered milieu. However, pathophysiological mechanisms related to poor oral health leading to adverse pregnancy outcomes need to be further elucidated.

Our study has some limitations. Firstly, the non-pregnant group consisted of a selected population of women working in the hospital and university who could have better oral knowledge of oral hygiene, and thus the results may not be generalizable to other populations. Secondly, we only investigated colonization of oral cavity by SM and LB rather than investigating the whole oral microbiome. Although these are the most important pathogens, the possible role of other microbes in causing pregnancy associated changes in oral cavity cannot be ignored. Furthermore, we did not perform clinical oral examination before saliva sampling. However, our study participants were healthy and none of them reported having any significant medical illness or oral health problems. Additionally, as our study had a cross-sectional design, the question of whether there are gestational-age-related serial changes in the oral bacterial milieu from beginning to the end of pregnancy remains unknown.

## Conclusion

Abundant bacterial colonization of oral cavity by both SM and LB was observed among healthy pregnant women during mid-pregnancy. Pregnancy appears to have an adverse impact on oral bacterial milieu as demonstrated by significantly increased colonization with SM together with higher OS levels and decreased TAC levels in the saliva. This emphasizes the importance of improved oral hygiene and provision of oral healthcare services during pregnancy.

## Data Availability

The dataset generated and/or analyzed during the current study are not publicly available due to concerns over participant confidentiality but are available from the corresponding author on reasonable request.

## Abbreviations

ABTS: 2,2′-azino-bis(3-ethylbenzothiazoline)-6-sulfonic acid diammonium salt
CFU: Colony forming unit
LB: *Lactobacillus*
MDA: Malondialdehyde
OD: Optic density
OS: Oxidative stress
ROS: Reactive oxygen species
SM: *Streptococcus mutans*
TBA: Thiobarbituric acid
TAC: Total anti-oxidative capacity
